# Access to public transportation and health facilities offering long-acting reversible contraceptives among residents of formal and informal settlements in two cities in Kenya

**DOI:** 10.1186/s12978-019-0828-0

**Published:** 2019-11-08

**Authors:** Veronica Escamilla, Lisa Calhoun, Norbert Odero, Ilene S. Speizer

**Affiliations:** 1 Carolina Population Center, University of North Carolina (UNC), Chapel Hill, USA; 2 Duke Global Health Institute, Duke University, Durham, USA; 3 Gillings School of Global Public Health, Department of Maternal and Child Health, UNC, Chapel Hill, USA

**Keywords:** Long-acting reversible contraceptive methods (LARC), Reproductive health, Public transportation access, Matatus, Health facility access

## Abstract

**Background:**

Despite improved health facility access relative to rural areas, distance and transportation remain barriers in some urban areas. Using household and facility data linked to residential and transportation geographic information we describe availability of health facilities offering long-acting reversible contraceptive (LARC) methods and measure access via matatus (privately owned mid-size vehicles providing public transport) in urban Kenya.

**Methods:**

Study data were collected by the Measurement, Learning and Evaluation (MLE) Project. Location information for clusters (2010) representative of city-level population were used to identify formal and informal settlement residents. We measured straight-line distances between clusters and facilities that participated in facility audits (2014) and offered LARCs. In Kisumu, we created a geographic database of matatu routes using Google Earth. In Nairobi, matatu route data were publicly available via the Digital Matatus Project. We measured straight-line distance between clusters and matatu stops on ‘direct’ routes (matatu routes with stop(s) ≤1 km from health facility offering LARCs). Facility and matatu access were compared by settlement status using descriptive statistics. We then used client exit interview data from a subset of facilities in Nairobi (*N* = 56) and Kisumu (*N* = 37) Kenya (2014) to examine the frequency of matatu use for facility visits.

**Results:**

There were 141 (Informal = 71; Formal = 70) study clusters in Nairoibi and 73 (Informal = 37; Formal = 36) in Kisumu. On average, residential clusters in both cities were located ≤1 km from a facility offering LARCs and ≤ 1 km from approximately three or more matatu stops on direct routes regardless of settlement status. Client exit interview data in Nairobi (*N* = 1602) and Kisumu (*N* = 1158) suggest that about 25% of women use matatus to visit health facilities. On average, women who utilized matatus travelled 30 min to the facility, with 5% travelling more than 1 hour. Matatu use increased with greater household wealth.

**Conclusions:**

Overall, formal and informal settlement clusters were within walking distance of a facility offering LARCs, and multiple matatu stops were accessible to get to further away facilities. This level of access will be beneficial as efforts to increase LARC use expand, but the role of wealth and transportation costs on access should be considered, especially among urban poor.

## Plain English summary

Distance and transportation remain barriers to health facility access in some urban areas. Using household and facility data linked to residential and transportation geographic information we describe availability of health facilities offering long-acting reversible contraceptive (LARC) methods and measure access via matatus (privately owned mid-size vehicles providing public transport) in urban Kenya. Location information for clusters (2010) representative of city-level population were used to identify formal and informal settlement residents. We measured distances between clusters and facilities that participated in facility audits (2014) and offered LARCs. In Kisumu, we created a geographic database of matatu routes using Google Earth. In Nairobi, these data were publicly available. We measured straight-line distance between clusters and matatu stops that were on ‘direct’ routes (matatu routes with stop(s) ≤1 km from facility offering LARCs). Using client exit interview data from health facilities in Nairobi and Kisumu, Kenya (2014), we examined matatu use for facility visits. On average, residential clusters in both cities were located ≤1 km from a facility offering LARCs and ≤ 1 km from approximately three or more matatu stops on direct routes regardless of settlement status. Client exit interview data in Nairobi and Kisumu suggest that about 25% of women use matatus to visit health facilities. On average, women who utilized matatus travelled 30 min to the health facility. Matatu use increases with greater household wealth. This level of access in Nairobi and Kisumu will be beneficial as efforts to increase LARC use expand, but effects of wealth and transportation costs on access must be considered.

## Background

Health outcomes and access to health services are on average, better in urban areas compared to rural areas, but heterogeneity exists within urban environments [1]. Urban poor living in informal settlements often face the challenge of insufficient access to quality health care options [2]. Urban poor women seeking family planning and child health services may have access to private health facilities within informal settlements but the facilities may not have a full array of services or family planning methods, and unlike the public sector where many services are free or inexpensive, the cost may be a burden [3, 4]. Public facilities are seldom located in poor neighborhoods or are located on the outskirts of the slums rather than in slums [3, 5]. In addition, public facilities are often overcrowded with long wait times, have stock outs of essential supplies, and are perceived to be of lower quality [4, 6–8]. As a result, women may have to travel some distance outside of their neighborhood to visit a private facility perceived to be higher quality [9], or to visit public facilities with free or reduced cost services [6]. Further, women who want to use long-acting reversible contraception (LARC) may need to travel further to a facility that offers LARCs and has higher perceived technical capability to offer LARCs [6].

It is well established that distance to a health facility can be a barrier for women seeking maternal, newborn, and child health (MNCH) services in rural sub-Saharan Africa [10–15]. Studies in rural Mozambique and Ghana also found that increased distance is associated with lower contraceptive use at the community and individual levels [16, 17]. In urban settings, with increased availability of health facilities, distance is often not considered a deterrent from seeking maternal health services but may remain a challenge for some. Women living in informal settlements in Nairobi, Kenya reported distance as a reason for selecting home delivery over hospital based delivery [18]. A study in urban Democratic Republic of Congo found that some women reported distance as a reason for not attending post-natal care consultations [19]. Distance was also found to have a negative impact on modern contraceptive use in urban Senegal [20]. Women cited travel as a primary reason for discontinuing modern contraceptive use in urban Niger and The Gambia [21], and women living in close proximity to a private clinic in urban Uganda were more likely to use modern contraception [22]. A study in Guatemala found higher modern contraceptive use among women living within 2 km of a high quality facility compared to women living further away [23]. Yet many of the studies that cite distance as a barrier to seeking health services do not utilize geographic information to fully understand this recognized barrier [11, 24].

Beyond distance, transportation access and associated costs present challenges for women seeking health services in rural and urban settings [18, 25–27]. Urban environments are distinct from rural areas in that there are often more forms of transportation available to the population. Forms of mass transit may exist such as public buses and trains, as well as private minibus taxis or motorcycle taxis [28, 29]. Individuals may also own cars, bicycles, or motorbikes. In many African cities, walking and public transportation are the main forms of transit, especially among the urban poor [30–32]. In Kenya, matatus, or privately owned mid-size vehicles that provide public transportation, are the most commonly used means of transportation in many large cities. At the moment, the government of Kenya does not have any other investments in public transportation besides the standard gauge railway system that connects two major cities. Other alternative modes of transportation such as larger country buses serve to connect larger cities and towns across the country. Matatus are not part of a formal transit system, and route changes are often developed by the matatu industry in response to demand [33]. Matatu routes in Nairobi loosely follow the former Kenya Bus Service transit system that is no longer available, and new routes have been added to reach the edge of the city, and in some instances connecting cities [34, 35]. While there are many transportation options in Kenya, residents of informal settlements face added challenges to accessing transportation. Many residents in informal settlements cannot afford matatu fees and must walk [36]. Informal settlement residents who can afford matatus also face challenges because poor road networks can impede matatus from entering informal settlements [37], requiring residents to walk outside of the settlement to access the matatu. Each of these transportation methods varies in terms of cost, time, and ease of movement within the city affecting access to health services.

The maternal and reproductive health service delivery environment in urban settings comprises public and private health facilities at multiple levels, including hospitals, health clinics, dispensaries and pharmacies. In Kenya, women can receive modern contraception at no cost from public health facilities [38]. Women can obtain LARCs from both public and private facilities, however implants and intrauterine devices (IUD) require a facility visit to a hospital or health clinic [39]. Shorter acting methods (e.g., injectables, pills, and condoms) have the flexibility of being obtained at a wider range of service delivery points, including pharmacies that sell prescription and non-prescription medicines, or even small shops selling other household goods [40]. While LARCs require a facility visit and may be more cumbersome to obtain than shorter acting methods, they are highly effective at preventing unplanned pregnancy and have high satisfaction rates among women of reproductive age, including adolescents [41–43]. Evidence also suggests that programs introducing LARCs are effective in both the private and public sectors, and are able to reach very low-income as well as higher-income women [44, 45]. In urban settings in Kenya, women obtain LARCs from a mix of public and private facilities. For example, among women living in Nairobi and Kisumu in 2014, public facilities were the primary source for implants, while private facilities were the primary source for IUDs [39]. Given that LARCs require a facility visit, accessing a facility that offers these methods may be a barrier to use; this requires careful consideration and deeper analysis.

The primary objective of this exploratory study was to describe the availability of facilities offering LARCs relative to the location of formal and informal settlements and matatu routes. We compared access between women living in formal and informal settlements because informal settlements often lack licensed and affordable health facilities as well as suitable road networks creating additional barriers for residents seeking family planning [3–5, 37]. We focus on two cities, Nairobi with available existing matatu route data, and Kisumu where we had the ability to generate matatu route data to better understand access in both large and mid-size urban areas. Existing studies often lack spatial data and are unable to examine geographical access [25]. Our unique dataset allows us to examine the spatial distribution of facilities offering LARCs relative to public transportation routes linked to the location of where a sample of women of reproductive age live. We focus on access to LARCs because they require a facility visit and there are growing efforts to introduce these methods in Kenya [46]. While we are not able to measure matatu use or capture current LARC use with this dataset, we are able to describe the availability of facilities offering LARCs and transportation resources relative to the location of clusters representing city-level population distribution as a representation of potential access. The secondary objective of this study was to examine matatu use among women seeking family planning services, using a separate dataset of client exit interviews from a subset of participating high-volume facilities. We are not able to examine matatu use by settlement status using this additional dataset, however we are able to examine differences in matatu use by wealth group of clients surveyed. Our exploratory study will help inform our understanding of public transportation access and travel burden for obtaining LARCs in areas where private transport options may be limited.

## Methods

### Study population

We analyzed primary data collected by the Measurement, Learning & Evaluation (MLE) Project. In Kenya, the objective of the MLE Project was to evaluate Tupange, one of the Bill & Melinda Gates funded Urban Reproductive Health Initiative programs, that was a comprehensive program with the goal of increasing modern contraceptive use in five urban sites in Kenya (Kakamega, Kisumu, Machakos, Mombasa, and Nairobi) from 2010 to 2014 (see Benson et al. [47] for additional MLE Project details).

Baseline data were collected in 2010 from a representative sample of women from the five program cities. A two-stage sampling design was used. Government enumeration areas (EA) designated as formal and informal settlements served as primary sampling units (PSU), and were randomly selected within each domain. Formal and informal settlement status was defined at the EA level by the Kenya National Bureau of Statistics during the 2009 Census and distinguishes between areas based on whether the majority of the households in the EA have property titles and official services [48, 49]. In the second stage, a household listing was undertaken in each selected PSU and then 30 households were randomly selected. All women aged 15–49 years in selected households were eligible for interview and invited to participate in the survey after providing informed consent. Weights were used to create representative samples from each city. Surveys captured use of maternal and child health and family planning services and information on which facility the woman went to obtain these services. Global Positioning System (GPS) coordinates of primary sampling unit (i.e. household cluster) centroids were also collected (See MLE baseline report [47] for details of survey and GPS data collection). This study utilizes data from the women’s survey to identify the universe of health facilities included in this analysis (described below), and cluster location information to examine citywide access to health facilities and matatu routes for a representative sample of women living in formal and informal settlements. All surveyed clusters were included in this analysis. For Nairobi, there were 141 (Informal = 71; Formal =70) study clusters included and for Kisumu the number was 73 (Informal = 37; Formal = 36). This study focuses on Nairobi and Kisumu due to availability of geographic data on transport routes in Nairobi and our capacity to generate a similar dataset in Kisumu.

### MLE facility data

A census of public health facilities including hospitals, health centers, and clinics was conducted in each city in 2014. In Kisumu, a census of all private facilities offering sexual and reproductive health services was also conducted. It was not possible to conduct a census of all private facilities in Nairobi given the high number of facilities. Therefore, we surveyed a sample of private facilities. Our sample of private facilities in Nairobi was constructed by compiling different sources of information. The list included all facilities where the Tupange program was working. In addition, during the 2010 household survey, women were asked to name the facility where they sought services for family planning, maternal health, and child health. We tabulated these responses at the cluster level, and then included the most frequently mentioned facilities in our facility list. Data collection in facilities included provider surveys, client exit interviews, and facility audits. Client exit interviews were only collected in high volume facilities to avoid having some facilities with a small number of exit interviews available.

Facility audits captured services offered, public/private status, and location information. We limited our analysis to health facilities that reported offering LARCs in 2014. In Nairobi, all 52 public sector facilities that were surveyed offered LARCs. We restricted our Nairobi analysis to include only public facilities because we have a census and can capture the current state of access to public facilities via matatus. We did not include private facilities in this analysis because we do not have a census, but rather a sample, and this could misrepresent actual availability of and access to private facilities offering LARCs via matatu routes in Nairobi. In Kisumu, all 18 public sector facilities that were surveyed offered LARCs. The private facility census in Kisumu identified 26 private facilities that offered LARCs. These data were then used to examine access to facilities offering LARCs via matatu routes.

### Matatu data

Matatu route data for Nairobi, Kenya were obtained from the Digital Matatus Project (http://www.digitalmatatus.com/map.html). Data include location information of matatu routes and stops associated with each route in Nairobi. As documented by Digital Matatus, data were collected by student team members who collected route data using handheld GPS units and mobile phones with GPS tracking capabilities while riding the matatus. Matatu stops were identified using students’ personal knowledge, signs or other forms of visual notation, and confirmation from frequent users or matatu operators. Data were downloaded and imported into a geographic information system (GIS) to identify routes with stops near health facilities offering LARCs and stops near clusters.

A similar geographic dataset of matatu routes in Kisumu did not exist so we created one using Google Earth Pro 7.3. Informed by local knowledge, we digitized matatu routes and matatu stops in Kisumu using satellite imagery (Satellite images: Google, DigitalGlobe). The satellite images for Kisumu were taken on 8/14/16 and 3/11/17. Digitized matatu routes and stops were then shown to team members with local knowledge of the field site and were edited accordingly. In addition, field assistants living in Kisumu rode local matatu routes to confirm designated stops.

### Geographical Measures

Euclidean (straight-line) distance between matatu stops and facilities offering LARCs was measured using ArcGIS v10.5. Previous studies found a decline in modern contraceptive use among women living beyond 2 km from a facility [17], and a decline in facility delivery among women living more than 1 km from a facility [12]. Therefore, we defined ‘direct’ matatu routes as routes with stops located within 1 km of these health facilities. We use a 1 km rather than a 2 km threshold to account for the fact that most women will also walk to the matatu stop from their home adding time to their trip. We then measured distance between household cluster locations and stops on direct routes, and identified the total number of direct routes with stops within 1 km of each cluster. We also calculated the total number of stops for any of these direct routes that were within 1 km of each cluster as a measure of access to the matatu routes. Distance was also measured between household cluster location and the nearest facility offering LARCs. The clusters in the sample provide a representation of women in the city and are not meant to be exhaustive of places of residence.

We generated additional distance measures between public hospitals and household clusters to identify clusters located beyond 5 km of a government hospital. We calculated this measure for public hospitals specifically because some women prefer obtaining family planning services at government hospitals [50]. A 5 km threshold was used because fewer public hospitals exist, and the Kenya Ministry of Health aims to have health service availability within 5 km of all residents [51]. Descriptive statistics were used to compare distance to health facilities and stops on direct routes by formal and informal settlement cluster status.

### Client exit interview data and measures

A total of 173 facilities were surveyed in Nairobi, of which 32% were selected and participated in client exit interviews. In Kisumu, 65 facilities were surveyed, of which 57% completed client exit interviews. For the client exit interviews, the objective was to include women visiting the facilities for both family planning services as well as for other maternal, newborn, and child health services; this latter group provided a perspective on access to integrated family planning services. Data collected from client exit interviews in 2014 were examined to better understand clients’ mode of transportation, specifically matatu use and travel time to higher volume facilities.

We constructed a categorical wealth index variable for women who participated in the high-volume facility exit interviews using principal components analysis [52]. The wealth index is a composite score of household asset ownership including mobile phone, TV, radio, computer, VCR, refrigerator, iron, fan, and gas cooker, and household structure characteristics including concrete roof, indoor toilet, piped water in dwelling, number of rooms, and electricity. We divided the wealth index into quintiles ranging from poorest (1) to richest (5). We present the sample distribution by wealth index for each city, because formal and informal settlement status was not captured as part of the client exit interview survey. We then created a binary variable representing poor (wealth index 1–2) and non-poor (wealth index 3–5) women and measured the difference between matatu use and walking by wealth using the Chi-square test. All analyses were conducted using Stata v14.0.

## Results

### Facility and matatu access

Location information based on the household survey from all selected clusters in Nairobi and Kisumu (Table 1) was used to measure access to matatus and health facilities that were representative of the city. By design, half of the clusters were located in informal settlements in both cities.

**Table 1:** Distance from study clusters to matatu stops on ‘direct’ routes and to health facilities offering LARCs by type of settlement (formal or informal) in Nairobi and Kisumu, Kenya

	Nairobi cluster level access to health facilities		Kisumu cluster level access to health facilities	
	Informal clusters *N* = 71	Formal clusters *N* = 70	Informal clusters *N* = 37	Formal clusters *N* = 36
Mean total direct routes^**£**^ within 1 km of cluster Mean total number of stops from direct routes located within 1 km of cluster	7.3 10.9	8.0 15.0*	1.6 2.8	3.3*** 3.6
Median distance to nearest stop on direct route, km (range) % clusters located within 1 km of stop	0.5 (0.1–1.4) 86.0	0.4 (0.1–12.9) 80.0	0.4 (0.1–2.9) 70.3	0.5 (0.1–5.6) 75.0
Median distance to nearest public facility offering LARC, km (range) % clusters beyond 5 km	0.9 (0.04–2.2) 0.0	0.8 (0.1–6.2) 2.9	1.1 (0.3–2.9) 0.0	0.8 (0.1–5.4) 2.8
Median distance to nearest public hospital offering LARC, km (range) % clusters beyond 5 km	3.6 (0.1–7.6) 40.9	2.6 (0.1–15.7) 21.4	1.8 (0.7–5.2) 2.7	1.5 (0.3–7.7) 19.4
Median distance to nearest private^**β**^ facility offering LARC, km (range) % clusters beyond 5 km	N/A	N/A	0.6 (0.1–3.2) 0.0	0.6 (0.1–6.0) 5.6
Median distance to nearest private^**β**^ hospital offering LARC, km (range) % clusters beyond 5 km	N/A	N/A	1.3 (0.3–3.2) 0.0	0.9 (0.1–7.0) 8.3

*p < 0.1; p*** < 0.01 for comparison between informal and formal settlements

^**£**^Direct routes defined as matatu routes with stops within 1 km of a public health facility offering long-acting reversible contraception (LARC)

^**β**^Distance measures to private facilities are not reported for Nairobi because a census of private facilities was not conducted

**Fig. 1:**
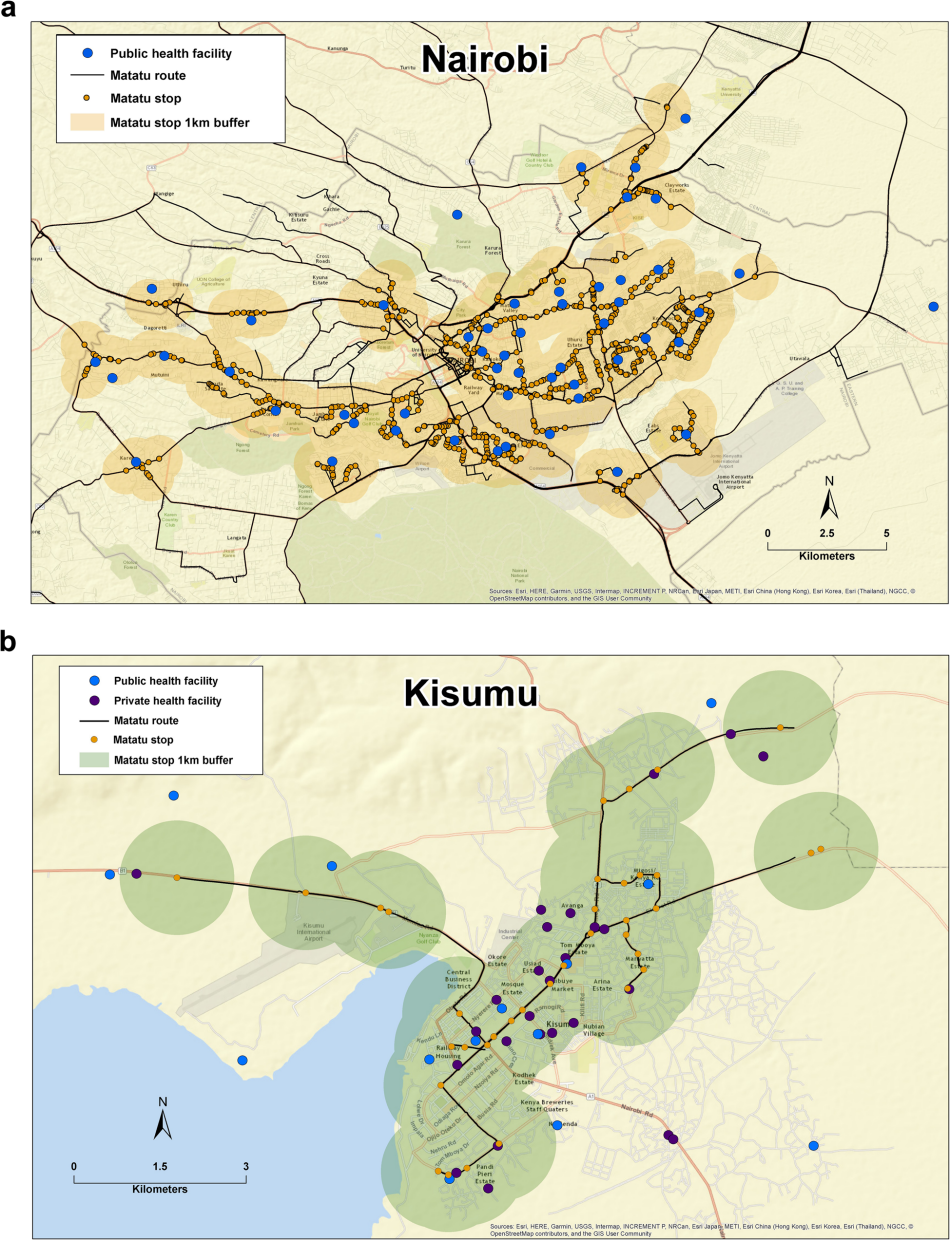
**a**-**b** Representation of citywide availability of health facilities that offer LARCs in relation to matatu stops; 1 km buffers surrounding matatu stops used to demonstrate distance between facilities and matatus in Nairobi (**a**) and Kisumu (**b**), Kenya. Note: Clusters are not shown to protect participant confidentiality

In Nairobi, a total of 127 matatu routes with stops within 1 km of a public facility offering LARCs were identified (Fig. 1a). On average, clusters in both formal and informal settlements were within 1 km of a minimum of 7 direct routes (Table 1). The average number of matatu stops on direct routes within 1 km of a household cluster was significantly higher for clusters located in formal settlements, however clusters in informal settlements still had access to nearly 11 stops. The median distance to any public facility offering LARCs was approximately 1 km for clusters in formal and informal settlements. Median distance to the nearest public hospital was slightly higher for clusters in informal settlements where twice as many clusters were further than 5 km compared to clusters in formal settlements (Table 1).

All seven matatu routes in Kisumu had stops within 1 km of a public facility offering LARCs (Fig. 1b). On average, clusters in formal settlements had greater access to direct routes and stops on direct routes within 1 km (Table 1). Median distance to public facilities offering LARCs was slightly greater for clusters in informal settlements compared to formal settlements. However, nearly 20% of clusters in formal settlements were beyond 5 km from a public hospital compared to 2.7% of clusters in informal settlements. The median distance between private facilities offering LARCs and clusters in both formal and informal settlements was ≤1 km. However, all clusters in informal settlements were within 5 km of a private hospital and 8.3% of clusters in formal settlements were beyond 5 km of a private hospital (Table 1).

### Facility exit interviews

Of the 56 facilities that completed exit interviews in Nairobi, 77% were public, including four public hospitals. The remaining were private facilities in Nairobi and included one private hospital. In Kisumu, 46% of the 37 facilities that participated in client exit interviews were public, including two public hospitals. The remaining facilities in Kisumu were private and included six private hospitals.

In Nairobi, client exit interviews took place with a total of 1602 women (Table 2). About 50% of the women in Nairobi were seeking family planning as their primary reason for visiting the facility, another 44% were seeking maternal or child health services, and the remaining sought curative services. In Kisumu, 32% of the 1158 women participating in exit interviews at facilities reported visiting for family planning, 30.5% sought maternal and child health related services, and the remaining 37.5% sought curative services. In both Nairobi and Kisumu, the median reported travel time to any facility was 20 min, apart from public hospitals where the median travel time was 30 min (not shown). While most women could reach a facility within 45 min, 12.9% of women in Nairobi travelled 50 min to several hours, and 13.6% of women in Kisumu travelled 60 to 180 min (not shown). A similar percentage of women in both cities took a matatu to the facility (26% in Nairobi and 20% in Kisumu), however a higher percentage of women in Nairobi reported walking (71% in Nairobi and 52% in Kisumu) (Table 2). In Kisumu, 11.3% of women also reported arriving to the facility using a motorcycle or scooter (not shown).

**Table 2:** Distribution of client responses to facility exit interviews stratified by city

	Nairobi	Kisumu
Total women participate in client exit interviews	1602	1158
Place of exit interview, *%*		
Public health clinic or hospital	76.2	50.5
Private health clinic or hospital	23.8	49.5
Reported reasons for facility visit, *%*		
Family planning	48.4	32.1
Maternal and child health services	43.7	30.5
Curative services	7.9	37.4
Percent report visiting facility closest to home	62.0	58.1
Percent report choosing facility because near home	56.2	52.5
Primary reasons for not visiting facility closest to home (among those not visiting closest), *N*^a^	606	481
Cost	35.8	16.0
Lack of services sought	11.4	25.0
Percent who rode matatu to facility	26.0	20.7
Percent who walked to facility	71.0	52.4
Wealth index^b^		
Poorest	13.3	29.6
Poor	24.6	14.2
Middle	21.9	16.8
Rich	22.7	16.2
Richest	17.5	23.2

^a^Full sample reporting not visiting facility closest to home (Nairobi, *N* = 699; Kisumu, *N* = 549); Number included for reasons not visiting closest facility smaller because it is restricted to the women who reported a reason for not visiting facility closest to home (drops those with missing information)

^b^Composite score of household asset ownership including mobile phone, TV, radio, computer, VCR, refrigerator, iron, fan, and gas cooker, and household structure characteristics including concrete roof, indoor toilet, piped water in dwelling, number of rooms, and electricity

The distribution of services sought among women who rode a matatu was similar to the full sample of women (Table 3). In Nairobi, nearly half of women reported seeking family planning, followed by 44.1% seeking maternal and child health services, and 10.3% seeking curative services. In Kisumu, among women who rode a matatu, 31.3% reported seeking family planning, 39.9% were seeking maternal and child health services, and 28.8% were seeking curative services. The distribution of services sought among women who did not ride a matatu was also similar to the full sample distribution in both Nairobi and Kisumu (not shown). In Nairobi, the majority of matatu riders were interviewed at a public facility and approximately one third of the women chose their facility because it was closest to home (Table 3). In Kisumu, the majority of matatu riders visited a private facility and few women selected a facility because it was closest to home (Table 3). Median travel time among matatu users was 30 min for all facility types in both cities.^1^ However, compared to women walking, women using matatus travelled on average 10 min longer than women who reported walking (not shown). The majority of women who rode matatus reached the facility within 45 min, but about 17% of women travelled one hour and about 5% of women travelled more than an hour in either city (not shown). In Nairobi, the distribution of the wealth index among matatu users was generally higher, with more than 50% of matatu users in the two highest wealth categories (Table 3). Matatu use was highest among women in the ‘richest’ category in Kisumu. Results from the Chi-square test show a significant difference in matatu use compared to walking between poor and non-poor women, such that women with higher wealth are more likely to ride a matatu in both Nairobi (Chi-square 47.16; *p* < 0.01) and Kisumu (Chi-square with ties 31.69; *p* < 0.01).

**Table 3:** Distribution of client responses to facility exit interviews among clients who used a matatu to get to the clinic stratified by city

	Nairobi	Kisumu
Total women participate in client exit interviews	417	240
Place of exit interview, *%*		
Public health clinic or hospital	84.4	33.3
Private health clinic or hospital	15.6	66.7
Reported reasons for facility visit, *%*		
Family planning	45.6	31.3
Maternal and child health services	44.1	39.9
Curative services	10.3	28.8
Percent report visiting facility closest to home	37.2	17.5
Percent report choosing facility because near home	29.3	12.1
Primary reasons for not visiting facility closest to home (among those not visiting closest), *N*^a^	261	197
Cost	26.1	13.2
Lack of services sought	13.0	27.4
Wealth index^b^		
Poorest	9.2	19.4
Poor	15.2	13.9
Middle	20.8	14.4
Rich	27.8	19.8
Richest	27.1	32.5

^a^Full sample reporting not visiting facility closest to home (Nairobi, *N* = 417; Kisumu, *N* = 240); Number included for reasons not visiting closest facility smaller because it is restricted to the women who reported a reason for not visiting facility closest to home (drops those with missing information)

^b^Composite score of household asset ownership including mobile phone, TV, radio, computer, VCR, refrigerator, iron, fan, and gas cooker, and household structure characteristics including concrete roof, indoor toilet, piped water in dwelling, number of rooms, and electricity

## Discussion

The level of access to public transportation among clusters located in informal settlements compared to formal settlements was unexpected. On average, clusters in both formal and informal settlements were within 1 km of multiple stops on direct routes. This could be due to local matatu associations creating new routes in response to demand. While matatus do not enter some informal settlements due to poor road networks, drivers normally stop in areas accessible to residents and stop in non-designated bus stops when they encounter or anticipate large numbers of commuters.

Facility access was also similar for clusters located in formal and informal settlements which was surprising but could be due to the growing public health sector [53]. On average, clusters are located within 1 km of the nearest public health facility offering LARCs in both cities regardless of settlement status. With the exception of two clusters (2.9%) in formal settlements in Nairobi, and one cluster (2.8%) in formal settlements in Kisumu, all clusters were within 5 km of a public health facility offering LARCs. This finding is similar to a study by Noor and colleagues [53] that found that the majority of the population in Kenya in both urban and rural areas was within 5 km of a public health facility. In Kisumu, clusters are also located within 1 km of the nearest private facility offering LARCs. These results suggest that in Nairobi and Kisumu, many women could walk to access family planning, supporting the high percentage of women who reported walking in exit interviews.

Hospital access differed between formal and informal settlements. In Nairobi, twice as many clusters in informal settlements compared to formal settlements were located beyond 5 km from a public hospital offering LARCs. This could be a challenge for women who prefer obtaining family planning services from public hospitals [50]. The opposite was observed in Kisumu. Nearly 20 % of clusters in formal settlements were located beyond 5 km from a public hospital offering LARCs compared to only 3 % of clusters in informal settlements. This contradicts the idea that urban poor live on the outskirts of cities [54], however wealthy people living in formal settlements may choose to live further away and pay the necessary transport costs to access services. In Kisumu, this may be because several estates with large populations and higher wealth have chosen to build formal settlements on the outskirts of the city in the last decade in response to the rapidly growing population.

A limitation of this exploratory study is that we are unable to examine the relationship between transportation and LARC use among clusters in formal and informal settlements as this was not a part of the MLE survey. Another limitation is that we used cluster location information captured from a representative household survey in 2010 and were therefore unable to present recent measures of modern contraceptive use at the cluster level. The 2010 data allowed us to present an overview of availability of facilities offering LARCs relative to cluster locations representative of the city-level population; these data were linked to recently available matatu information to develop a general understanding of the current infrastructure in Kenya and Kisumu. The findings here can inform future studies that consider facility and transportation factors when measuring current LARC use and access.

While we were unable to measure the relationship between matatu use and LARC use, our client exit interview data provides some insight to matatu use among women seeking family planning in urban settings. Our findings demonstrate that women in Nairobi and Kisumu are using public transportation to visit health facilities, however walking is the primary method of transportation. While the majority of the population walks to health facilities, we see that about a quarter of women are taking matatus when seeking family planning and other maternal and child health services, including child immunization and antenatal care. Matatu use was higher among individuals with greater wealth in both cities; however, we were unable to make this comparison by formal and informal settlement status because that information was not obtained during exit interviews. Our results however support prior research in Nairobi that found slum residents with higher wealth were more likely to afford and use matatus compared to poor slum residents [36]. Travel times were similar for matatu users in both Nairobi and Kisumu, and on average slightly longer than travel times for women walking to facilities. It is possible that women are willing to take matatus despite longer travel times if they have the means available. Women may also choose to take matatus because of convenience or need due to difficulty walking or the type of service sought, or to avoid walking in unsafe areas.

We were unable to examine the role of cost on transportation use among our sample because this information was not collected during the exit interviews. Another limitation to note is the absence of matatu fare information. We were unable to collect this information because fares can vary based on the route, time of day, and weather. Further, we did not capture information on employment status. However, asset data collected from exit interviews serve as a proxy for wealth status and suggest that women with higher wealth use public transport more often than women with lower wealth. Future research should consider transportation costs and income when examining access and uptake of LARCs among the urban poor as efforts to increase LARC use continue.

To our knowledge, our study is the first to examine matatu access in relation to family planning accessibility. Using a unique public dataset for Nairobi, and generating our own matatu database for Kisumu using satellite imagery, we provide an example for future studies examining transportation and health service access in similar urban settings. A strength of our study is the availability of geographic data for household clusters and facilities that allow us to measure access via distance to facilities and public transportation. A limitation to consider is our use of straight-line distance rather than path distance in the absence of both road network information for informal settlements and data on transfers along matatu routes. While the straight-line distance measures do not capture time estimates for walking or driving, they provide a measure of varying access to facilities and matatu stops.

Recent increases in the adoption of LARCs in Kenya [45] and expanding efforts to promote LARCs could benefit from the accessibility of public transportation for women in both formal and informal settlements. Access could change with rapid urbanization, and it will be important to have a facility and transportation infrastructure that can accommodate the growing population.

## Conclusion

Access to matatus and health facilities offering LARCs is similar for both formal and informal settlements in Nairobi and Kisumu. This level of access will be beneficial as efforts to increase LARC use expand, requiring more women to visit health facilities, rather than pharmacies, to obtain a family planning method. Of note, matatu use was higher among women with greater wealth and walking remains the main form of transportation. Therefore, it will also be important to consider the role of transportation costs and wealth to accommodate the need for increased health facility visits for LARC use, especially among the urban poor. Future work should also measure the direct relationship between matatu and LARC use to better understand the role of transportation accessibility and use on modern contraceptive use.

## Data Availability

The survey instruments used to generate the data in this study are publicly available. Please see: https://dataverse.unc.edu/dataverse/mle_kenya The Nairobi matatu route data are available from the Digital Matatus Project. Please see: http://www.digitalmatatus.com/map.html The cluster location data that support the findings of this study are not publicly available due to them containing information that could compromise research participant privacy/consent.

## Abbreviations

EA: enumeration area
GIS: geographic information system
GPS: global positioning system
IUD: intrauterine device
LARC: long-acting reversible contraception
MLE: Measurement, Learning & Evaluation
MNCH: maternal, newborn, and child health
PSU: primary sampling unit

## Footnotes

1 Median distance to private hospital not computed in Nairobi because only one matatu user interviewed at a private hospital; reported travel time was 60 min.
